# Cardiac Symptoms of Chorea

**Published:** 1882-09

**Authors:** 


### Selections.

---

### Cardiac Symptoms of Chorea.

Dr. O. Sturges (*Brain*, July, 1881) summarizes the several factors of the heart symptoms thus: 1. In the course of the chorea of childhood the heart's action is apt to become irregular or uneven, and its first sound to be followed by apex-murmur, which is variable in pitch, influenced by posture, seldom audible in the axilla or at the angle of the scapula, and which disappears along with or shortly after the chorea, the heart and the circulation suffering no injury. 2. This liability on the part of the heart to what, from its signs, would seem to be a functional disturbance, is independent of the violence or method of the chorea, but dependent upon the age of the patient, the younger children being most, and the elder least, liable; while beyond childhood there is little, if any, liability of the kind. 3. These heart signs of chorea—acute rheumatism being excluded—give rise, as a general rule, to no symptoms whatever affecting the health or comfort of the child. They make no apparent difference to the prospects of recovery, or to the structural integrity of the heart. Nevertheless, choreic children having this murmur and happening to die either with, or shortly after recovery from the chorea, very commonly exhibit a beading of recent lymph on the mitral valves. Such, he says, are the chief statements which statistics seem to warrant. To these he adds another, which, so far as he knows, has never been statistically recorded, but which no one will gainsay. It is, indeed, the most constant of all the heart symptoms of chorea, and met with at a later age than the rest. He refers to the acceleration of the heart and pulse.—*Amer. Jour. Med. Sci.*, Jan., 1882.

